# RINL, Guanine Nucleotide Exchange Factor Rab5-Subfamily, Is Involved in the EphA8-Degradation Pathway with Odin

**DOI:** 10.1371/journal.pone.0030575

**Published:** 2012-01-23

**Authors:** Hiroaki Kajiho, Shinichi Fukushima, Kenji Kontani, Toshiaki Katada

**Affiliations:** Department of Physiological Chemistry, Graduate School of Pharmaceutical Sciences, University of Tokyo, Tokyo, Japan; Institut Européen de Chimie et Biologie, France

## Abstract

The Rab family of small guanosine triphosphatases (GTPases) plays a vital role in membrane trafficking. Its active GTP-bound state is driven by guanine nucleotide-exchange factors (GEFs). Ras and Rab interactor (or Ras interaction/interference)-like (RINL), which contains a conserved VPS9 domain critical for GEF action, was recently identified as a new Rab5 subfamily GEF *in vitro*. However, its detailed function and interacting molecules have not yet been fully elucidated. Here we found that RINL has GEF activity for the Rab5 subfamily proteins by measuring their GTP-bound forms in cultured cells. We also found that RINL interacts with odin, a member of the ankyrin-repeat and sterile-alpha motif (SAM) domain-containing (Anks) protein family. In addition, the Eph tyrosine kinase receptor EphA8 formed a ternary complex with both RINL and odin. Interestingly, RINL expression in cultured cells reduced EphA8 levels in a manner dependent on both its GEF activity and interaction with odin. In addition, knockdown of RINL increased EphA8 level in HeLa cells. Our findings suggest that RINL, as a GEF for Rab5 subfamily, is implicated in the EphA8-degradation pathway via its interaction with odin.

## Introduction

Rab guanosine triphosphatases (GTPases) play pivotal roles in intracellular membrane trafficking. At present, more than 60 members have been identified; they are localized in distinct intracellular compartments and regulate intracellular transport specifically [1], [2]. Rab5, which is the most thoroughly characterized member of this family, is a key regulator of endocytosis, endosome fusion, and endosome trafficking [3]. Like other Rab GTPases, the transition from an inactive state [guanosine diphosphate (GDP)-Rab5] to an active state (GTP-Rab5) is mediated by guanine nucleotide exchange factors (GEFs). To date, many Rab5 GEFs have been identified and extensively analyzed [4]. All share a highly conserved vacuolar protein sorting 9 (VPS9) domain, which is required for bindings to, and nucleotide exchange on, Rab5 proteins. Rabex-5 is a VPS9 domain-containing protein that shows GEF activity for Rab5 and Rab21 GTPases; the VPS9 domain structure of Rabex-5 has been determined by X-ray analysis, and four amino acids (i.e., D, P, Y, and T) have been shown to be critical for its GEF activity [5].

The Ras and Rab interactor (or Ras interaction/interference, RIN) family proteins, composed of RIN1–3, also have a VPS9 domain and function as Rab5 GEFs [6]–[8]. Uniquely, RIN proteins contain many functional domains, including Src homology 2 (SH2), proline-rich (PR), RIN family homology (RH), and Ras association (RA) domains [6]. Previous studies have shown that RIN1 interacts with various receptor tyrosine kinases (RTKs), including epidermal growth factor receptor, platelet-derived growth factor receptor, and EphA4 receptor [9], [10]. RIN2 interacts with the HGF receptor [11], and RIN proteins generally regulate the membrane trafficking and degradation of RTKs. We previously identified RIN3 as a Rab5-GEF, and showed that tyrosine phosphorylation signals induce the translocation of cytoplasmic RIN3 to Rab5-positive early endocytic vesicles [6], [12]. We also showed that the RH domain is necessary and sufficient for the interaction between RIN3 and Rab5 proteins [12]. In addition, recently we reported that RIN3 specifically acts as a GEF for Rab31, one of the Rab5 subfamily proteins (i.e., Rab5, Rab21, Rab22, and Rab31) [13]. While the functions of RIN1–3 have been elucidated, RIN-like (RINL) was recently identified as a protein with high similarity to RIN proteins [14]. RINL is ubiquitously expressed with its highest expression in lymphoid organs, and exhibits GEF activity for Rab5 and Rab22 GTPases. However, detailed analysis about the biochemical activity of RINL or the identification of its interaction molecules has not yet been performed. In the present study, we evaluated the broad GEF activity of RINL for Rab5 subfamily proteins both using recombinant proteins and in mammalian cells and showed that RINL activated Rab5 proteins via its VPS9 domain. Moreover, we identified odin, a member of the ankyrin-repeat and sterile-alpha motif (SAM) domain-containing (Anks) protein family, as a molecule that interacts with RINL. Furthermore, RINL bound to EphA8 via odin and reduced EphA8 levels in a manner dependent on its GEF activity. These findings suggest that as a GEF activator of Rab5 proteins, RINL is implicated in the degradation of EphA8 via its interaction with odin.

## Results

### RINL stimulates the formation of GTP-bound Rab5 in intact cells

**Figure 1:**
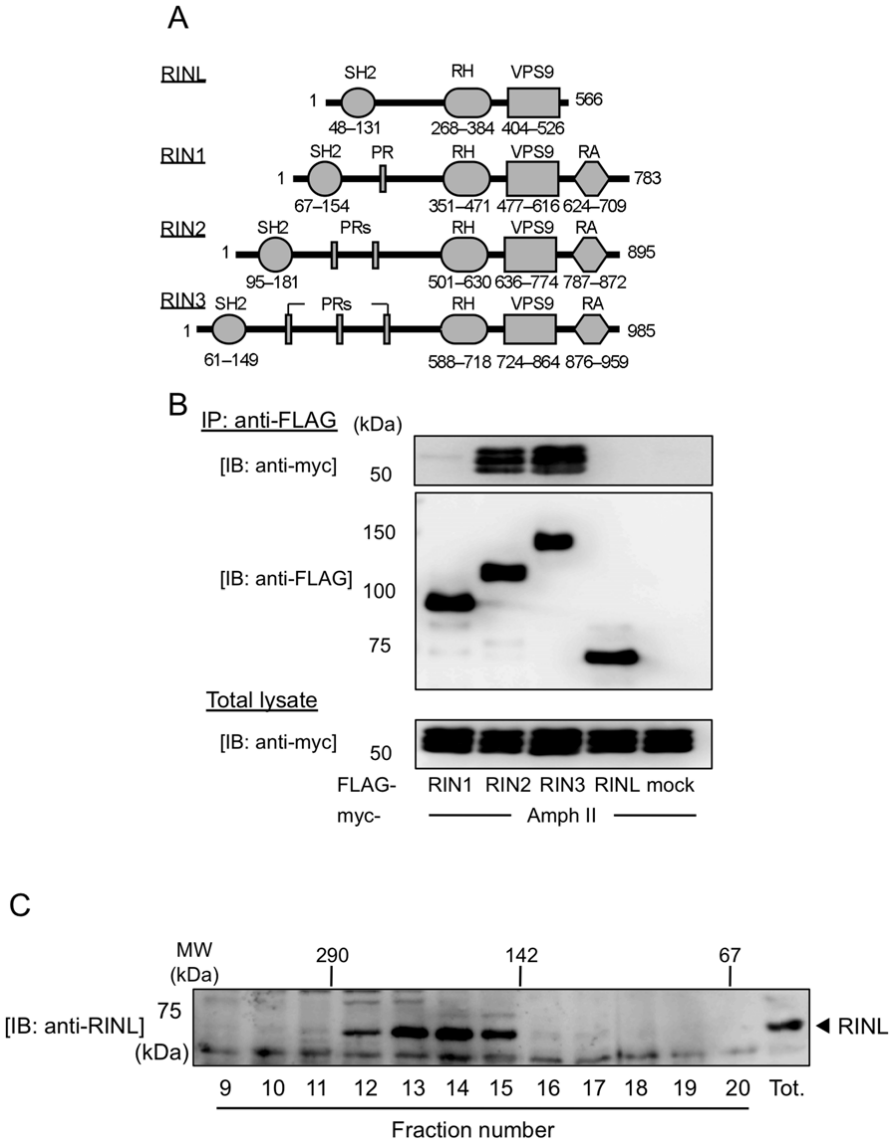
Structure of RINL. (A) Diagram of the structural features of RIN family members. The lower numbers represent the amino acid residues. (B) FLAG-RIN1, RIN2, RIN3, and RINL were transiently co-transfected with myc-amphiphysin II (amph II) into HEK293T cells. Cells lysates were immunoprecipitated with anti-FLAG antibody, followed by immunoblotting with anti-myc and anti-FLAG antibodies. Total lysates were immunoblotted with anti-myc antibody. (C) Cell lysates from HEK293T cells were applied to a Superdex 200 Prep Grade gel filtration column. The elution position was compared with those of the globular size markers (upper panel). The fractions (0.5 ml) eluted from the column and total lysate (tot.) were analyzed by SDS-PAGE, and proteins were immunoblotted with anti-RINL antibody.

RINL was identified as a protein with high similarity to RIN family proteins, including RIN1, 2, and 3 [6]–[8]. Although RINL shares SH2, RH, and VPS9 domains with other RIN family members, it lacks PR and RA domains (Fig. 1A). Therefore, RINL did not interact with amphiphysin II, which associates with RIN2 and RIN3 by their PR domains [6] (Fig. 1B).

Since RIN2 and RIN3 have been shown to function as tetramers composed of anti-parallel linkages of two parallel dimers [7] (data not shown), we investigated whether RINL forms homo-multimeric complexes in mammalian cells. Cell lysate from HEK293T cells were applied to a gel filtration column; fractions eluted from the column were analyzed by sodium dodecyl sulfate polyacrylamide gel electrophoresis (SDS-PAGE) and immunoblot by anti-RINL antibody. Endogenous RINL eluted from the column as a 200-kDa protein (Fig. 1C). Since the molecular weight of RINL is about 72,000, these results indicate that RINL does not appear to form a tetramer but exist as a multimer or a complex with other proteins.

Quite recently, RINL was reported to act as a GEF for both Rab5 and Rab22 GTPases *in vitro*, stimulating the dissociation rate of GDP from these GTPases [14]. We confirmed the GEF activity of RINL for Rab5 subfamily proteins by measuring the formation of GTP-bound forms of these proteins (Figure S1). Recombinant RINL proteins purified from baculovirus-infected Sf9 cells markedly accelerated [^35^S]GTPgammaS binding to Rab5a, 5b, and, 5c proteins as did Rabex-5. Rabex-5 strongly, as opposed to RINL, accelerated GTPgammaS binding to Rab21. In contrast, RINL accelerated GTPgammaS binding to Rab22 and Rab31. We also found that RIN3 markedly and RIN2 weakly exerted GEF activities for Rab22 and Rab31; however, RIN1 and Rabex-5 did not (Figure S2) [13].

**Figure 2:**
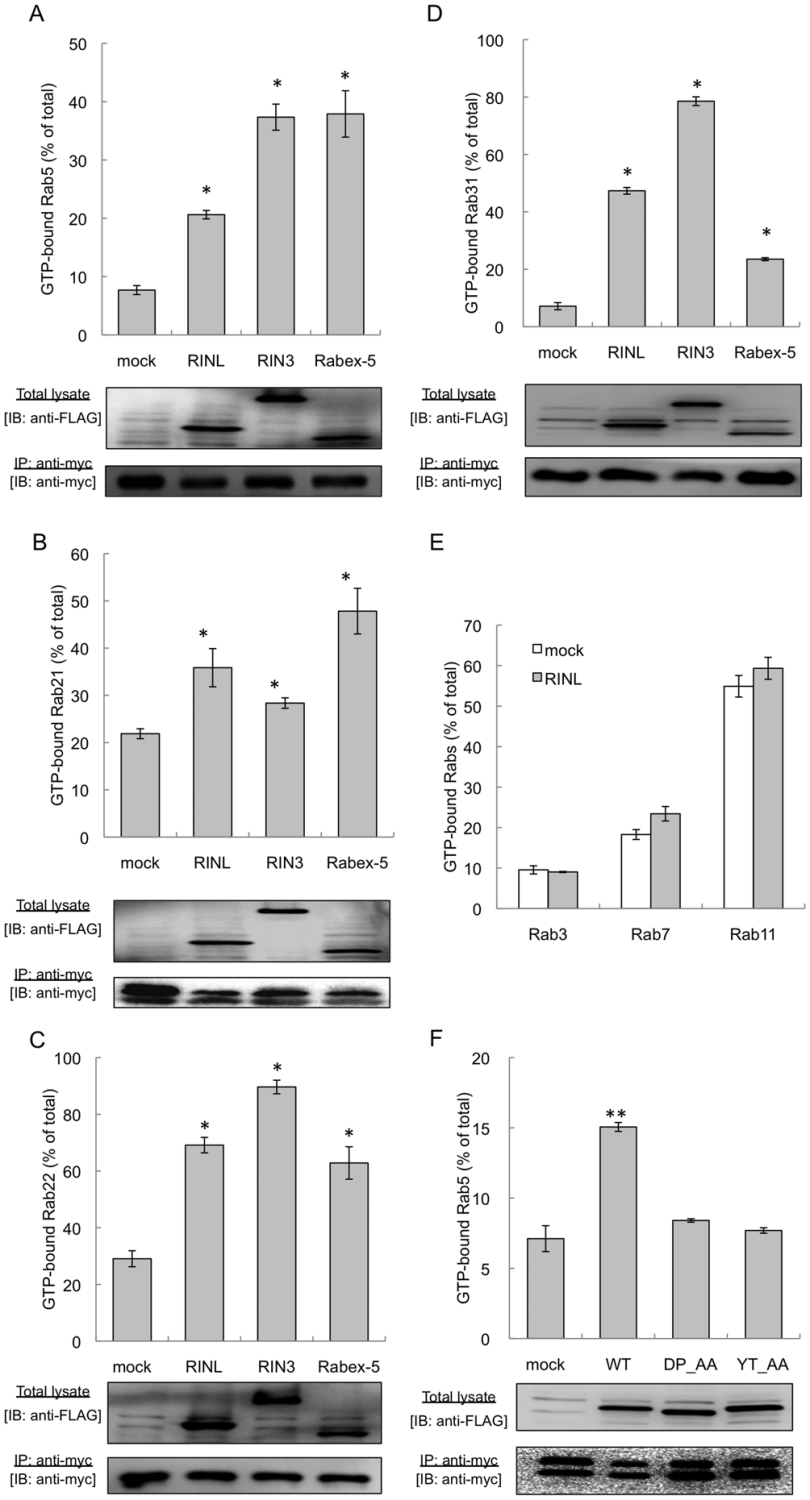
GEF activity of RINL for Rab5 subfamily proteins. (A–D) HEK293T cells expressing myc-Rab5b (A), Rab21 (B), Rab22 (C), or Rab31 (D) and FLAG-mock, RINL, RIN3, or Rabex-5 were metabolically radiolabeled with ^32^P_i_ for 4 hours. Myc-Rab5 subfamily proteins were immunoprecipitated with an anti-myc monoclonal antibody, and nucleotides associating with each Rab protein were separated by thin-layer chromatography. The radioactivity of GTP and GDP was quantified, and the percentages (%) of each GTP-bound Rab are shown. Total lysates (bottom) and immunoprecipitated samples (middle) from the radiolabeled cells were separated by SDS-PAGE and immunoblotted with anti-FLAG and anti-myc antibodies, respectively. *p<0.05 vs. mock-transfected cells. (E) Myc-Rab3a, 7a, or 11a was co-transfected with FLAG-mock or RINL into HEK293T cells. The percentages of each GTP-bound Rab member in the metabolically radiolabeled cells are shown as described in (A). (F) Myc-Rab5b was co-transfected with wild type (WT), or the DP_AA or YT_AA mutant of FLAG-RINL into HEK293T cells. The percentages of GTP-Rab5b in the metabolically radiolabeled cells are shown as described in (A). Total lysates (bottom) and immunoprecipitated samples (middle) from the radiolabeled cells were separated by SDS-PAGE and immunoblotted with anti-FLAG and anti-myc antibodies, respectively. All data were obtained from more than three independent experiments and are shown as the mean ± S.E. (error bars). **p<0.01 vs. mock-transfected cells.

We next investigated the GEF activity of RINL for Rab5 subfamily proteins in intact cells. Approximately 8% of Rab5b was present in a GTP-bound form in mock-transfected cells, and the expression of either RIN3 or Rabex-5 increased the formation of GTP-Rab5b effectively (Fig. 2A). The expression of RINL also increased GTP-Rab5b, but less effectively. We applied the same assay to other Rab5 subfamily proteins. RINL significantly increased GTP-Rab21, GTP-Rab22, and GTP-Rab31 formation, but less effectively than did either Rabex-5 or RIN3 (Fig. 2B–D). In contrast, RINL did not show any significant GEF activity for Rab3a, Rab7a, or Rab11a (Fig. 2E). These results show that RINL moderately stimulates the formation of GTP-Rab5 subfamily proteins in mammalian cells.

Since the four amino acids in the VPS9 domain, which are critical for the GEF activity of Rabex-5 [5], are conserved in RINL, we generated two RINL mutants with reduced GEF activity; RINL/D453A/P457A (DP_AA) and RINL/Y494A/T497A (YT_AA). We found that RINL/WT interacted with dominant-negative Rab5b/S34N using the yeast two-hybrid system as reported previously [14], while either RINL/DP_AA or YT_AA reduced this interaction (data not shown). Furthermore, the increase in GTP-Rab5b after co-expression of RINL was completely abolished by mutations of the VPS9 domain (Fig. 2F). These results clearly indicate that RINL exerts GEF activity for Rab5 subfamily proteins via its VPS9 domain.

### Identification of odin as a RINL-binding protein

**Figure 3:**
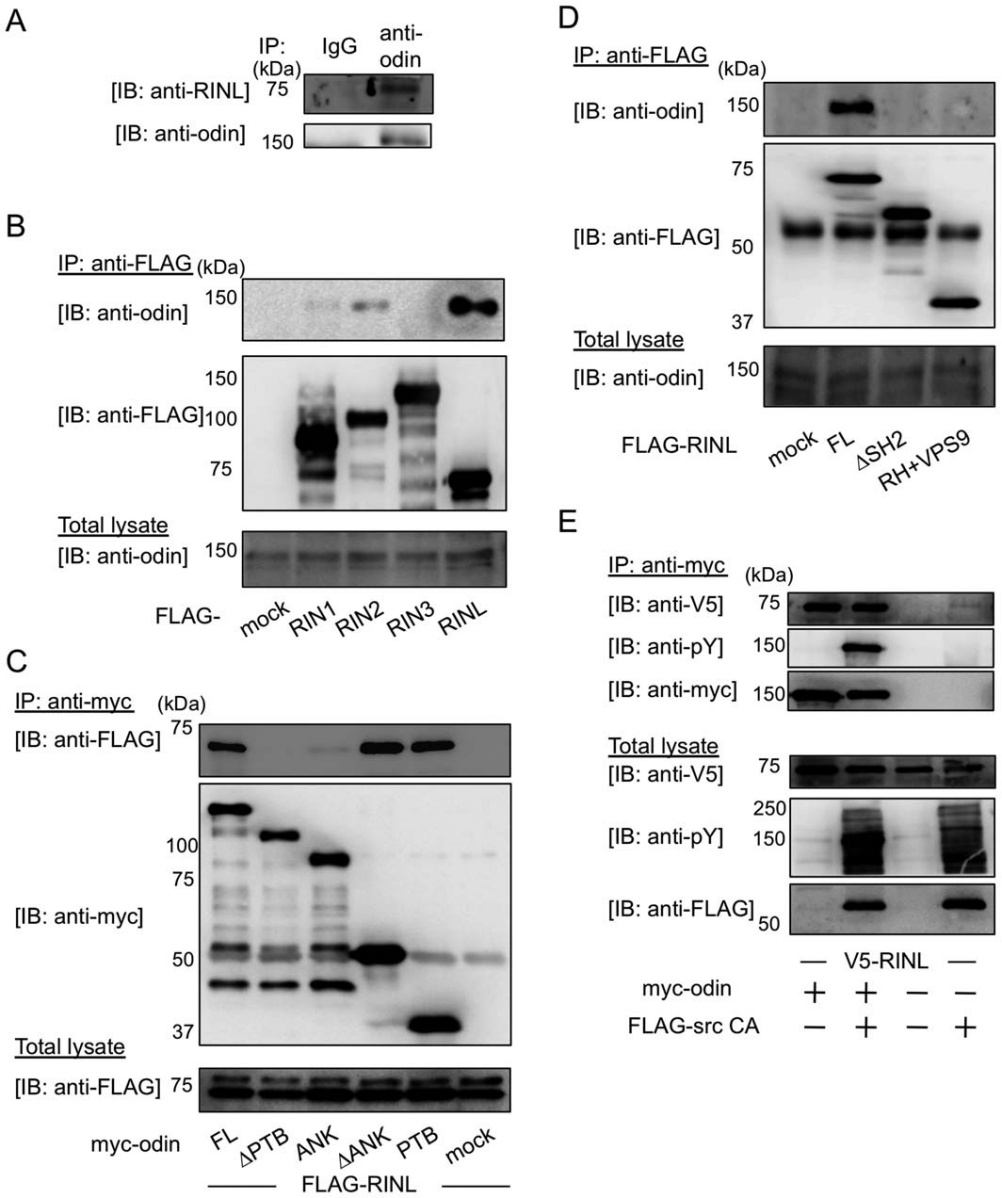
Identification of odin/Anks1a as an interacting molecule with RINL. (A) HeLa cell lysates were immunoprecipitated with normal rat IgG or anti-odin antibody, followed by immunoblotting with antibodies as indicated. (B) FLAG-RIN family or FLAG-mock were transfected into HEK293T cells. Cells lysates were immunoprecipitated with anti-FLAG antibody, followed by immunoblotting with antibodies as indicated. (C) FLAG-RINL and the indicated deletion mutants of myc-odin were transiently transfected into HEK293T cells. Cells lysates were immunoprecipitated with anti-myc antibody, followed by immunoblotting with antibodies as shown. (D) The indicated deletion mutants of FLAG-RINL were transiently transfected into HEK293T cells. Cells lysates were immunoprecipitated with anti-FLAG antibody, followed by immunoblotting with antibodies as indicated. (E) Myc-odin and V5-RINL were co-transfected with FLAG-tagged constitutively active (CA, lanes 2 and 4) or mock (lanes 1 and 3) into HEK293T cells. Cell lysates were immunoprecipitated with anti-myc antibody, followed by immunoblotting with antibodies as indicated. Aliquots of total lysates were also immunoblotted with antibodies as indicated.

To uncover the function of RINL, we further searched for RINL-binding proteins using the yeast two-hybrid system. A mouse brain cDNA library was screened with full-length RINL as bait. Screening of 3.5×10^5^ transformants yielded seventeen positive clones that strongly interacted with RINL. One was composed of a cDNA encoding a partial sequence of odin. The isolated odin clone corresponded to amino acids 583–1150 (Figure S3). Because odin belongs to the Anks protein family, it is also called Anks1a. Odin/Anks1a possesses a phosphotyrosine-independent Dab-like phosphotyrosine-binding (PTB) domain in its C-terminal region [15]. We investigated whether RINL associates with odin in mammalian cells at endogenous level. By using anti-odin and RINL antibodies, we found that endogenous odin and RINL are co-immunoprecipitated in HeLa cells (Fig. 3A). Next we examined the specificity of the interaction. When FLAG-RIN was expressed in HEK293T cells, RINL strongly interacted with endogenous odin, while RIN1 and RIN2 only weakly bound and RIN3 did not bind (Fig. 3B). We also found that RINL/DP_AA and YT_AA, GEF deficient mutants of RINL, also interacted with endogenous odin, though RINL/YT_AA bound moderately (Figure S4).

To identify the interacting regions between RINL and odin, a number of deletion mutants of these proteins were generated (Figure S5). Myc-tagged wild type and deletion mutants of odin were co-transfected with FLAG-RINL into HEK293T cells, and the lysates were immunoprecipitated with anti-myc antibody. The mutants containing the PTB domain bound RINL, but those lacking the PTB domain did not (Fig. 3C), clearly showing that the PTB domain is required and sufficient for the interaction of odin with RINL. Similar assays were applied to RINL, and identified that the SH2 domain of RINL is required for its interaction with odin (Fig. 3D). The SH2 domain generally recognizes and interacts with phosphorylated tyrosine residues, and odin has been reported to be tyrosine phosphorylated by Src family kinases [16]. However, when odin was phosphorylated by co-expression with constitutively active Src, odin interacted with RINL as strong as the non-phosphorylated form did (Fig. 3E). These results indicate that the odin interacts with RINL regardless of its tyrosine phosphorylation state.

### RINL forms a ternary complex with odin and EphA8, and RINL affects the degradation of EphA8 receptor

**Figure 4:**
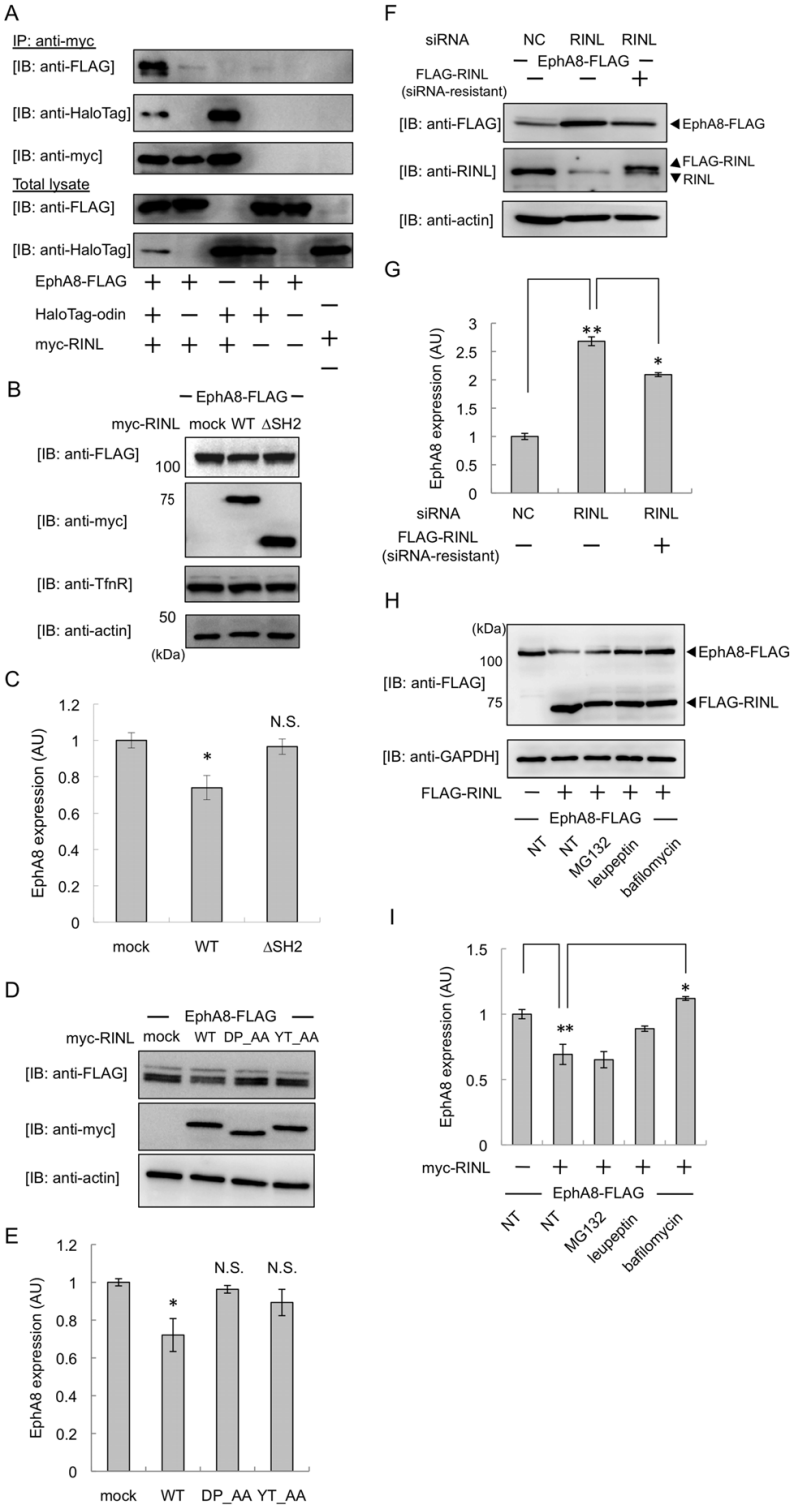
RINL forms a ternary complex with odin and EphA8, and RINL affects the degradation of the EphA8 receptor. (A) HEK293T cells were co-transfected with EphA8-FLAG, HaloTag-odin, and myc-RINL (+) or mock (−) plasmids as indicated, and cell lysates were immunoprecipitated with anti-myc antibody. Immunoprecipitated fractions and total lysates were immunoblotted with antibodies as indicated. (B and C) HeLa cells were transfected with EphA8-FLAG and myc-RINL or mock plasmids, and total lysates were immunoblotted with antibodies as indicated. ΔSH2; SH2 domain-deleted mutant. The data obtained from three independent experiments are shown (C) as the mean ± S.E. (error bars). *, p<0.05 vs. mock-transfected cells. N.S., not significant. (D and E) HEK293T cells were transfected with EphA8-FLAG and myc-RINL or mock plasmids, and total lysates were immunoblotted with antibodies as indicated. WT; wild type. The data obtained from three independent experiments are shown (E) as the mean ± S.E. (error bars). *, p<0.05 vs. mock-transfected cells. (F and G) HeLa cells were transfected with 30 pmol scrambled negative control (NC) or RINL-specific siRNA. 24 hours after the transfection, these cells were transfected with EphA8-FLAG and siRNA-resistant FLAG-RINL, and incubated for 48 hours. Total proteins from the cell lysates were subjected to SDS-PAGE and immunoblotted (IB) with antibodies as indicated. The data obtained from three independent experiments are shown (G) as the mean ± S.E. (error bars). **, p<0.01 vs. NC-transfected cells. *, p<0.05 vs. siRNA-transfected cells with FLAG-mock plasmid transfection. (H and I) HeLa cells were transfected with EphA8-FLAG and FLAG-RINL (+, lanes 2–5) or mock plasmids (−, lane 1), and total lysates were immunoblotted with antibodies as indicated. These cells were non-treated (NT, lanes 1 and 2), or treated with MG132 (20 µM, lane 3), leupeptin (100 µg/ml, lane 4), or bafilomycin (200 nM, lane 5) for 3 hours. Total lysates were immunoblotted with antibodies as indicated. The data obtained from three independent experiments are shown (I) as the mean ± S.E. (error bars). **, p<0.01 vs. mock-transfected cells. *, p<0.05 vs. non-treatment cells transfected with RINL.

It has been reported that odin interacts with a member of the Eph-receptor family, EphA8 [17], which we confirmed (data not shown). To investigate whether RINL forms a ternary complex with odin and EphA8 or RINL interacts odin alone, HEK293T cells were co-transfected with myc-RINL, HaloTag-odin, and EphA8-FLAG or their mock plasmids. The lysates from these transfected cells were immunoprecipitated with anti-myc antibody. RINL interacted with EphA8 in an odin-dependent manner (Fig. 4A), indicating that RINL forms a ternary complex with both odin and EphA8.

RIN proteins have been implicated in endocytosis of tyrosine kinase receptors, and RIN1 specifically regulates EphA4 signaling by promoting its endocytosis [10]. Since odin has been shown to protect EphA8 from degradation [18], we supposed that RINL might be involved in this degradation process. For this purpose, HeLa cells were co-transfected with myc-RINL and EphA8-FLAG. We found that EphA8 levels in the cell lysates were significantly reduced by RINL expression (Fig. 4B and C), while endogenous transferrin receptor levels were unaltered. The expression of RINLΔSH2, a mutant lacking the SH2 domain (Fig. 3C), did not reduce EphA8 levels significantly. This result suggests that the interaction between RINL and odin might be important for the degradation of EphA8. Furthermore, we found that the Rab5 GEF activity-defective mutants RINL/DP_AA and RINL/YT_AA did not significantly affect EphA8 levels (Fig. 4D and E), indicating that RINL expression promotes EphA8 degradation in a GEF activity-dependent manner. To eliminate the possibility that transient co-transfection of expression plasmids affects EphA8 levels, similar assays were used in Neuro2a cells stably expressing EphA8-HA. We found that RINL expression induced the degradation of EphA8 as well (Figure S6). To verify that RINL is involved in the degradation pathway of EphA8, we knocked down RINL in HeLa cells through transfection of a specific small interfering RNA (siRNA). Western blot analysis revealed that endogenous RINL was effectively reduced (Fig. 4F, middle panel). As expected, EphA8 level significantly increased by depletion of RINL (Fig. 4F and G), consistent with the ability of RINL to stimulate the degradation of EphA8. Moreover, the increase in EphA8 with RINL-siRNA was significantly rescued by expression of the siRNA-resistant RINL (Fig. 4F, third lane). To identify the EphA8 degradation pathway induced by RINL, we incubated RINL-expressing cells with the specific lysosomal inhibitor leupeptin, bafilomycin, and the proteasomal inhibitor MG132. Bafilomycin significantly, and leupeptin partially blocked the degradation of EphA8 by RINL (Fig. 4H and I), but MG132 did not. These results suggest that EphA8 is degraded in the lysosomal pathway by the expression of RINL.

## Discussion

To date, the VPS9 domain, which is a hallmark of Rab5 subfamily protein GEFs, has been found in many proteins, including RIN family members (RIN1–3), Vps9p, Rabex-5, ALS2/Alsin, Varp, and Gapex-5/RAP6/RME-6 [6]–[8], [19]–[23]. In the present study, we found that RINL activates Rab5 subfamily proteins in GEF assays *in vitro*. Moreover, we identified odin as an interacting molecule with RINL and showed that RINL is involved in EphA8 degradation of EphA8 via its interaction with odin.

While RINL significantly increased GTP-Rab21 in HEK293T cells (Fig. 2B), purified RINL protein weakly accelerated GTPgammaS binding to Rab21 *in vitro* (Figure S1). Similarly, Rabex-5 has been reported to exhibit 100-fold lower GEF activity for Rab22 than for Rab5 and Rab21 *in vitro* [5], increasing GTP-Rab22 levels in mammalian cells (Fig. 2C). These results indicate that RINL and Rabex-5 may require some cofactors to activate Rab21 and Rab22, respectively.

RINL exhibited moderate GEF activity for Rab5, Rab21, Rab22, and Rab31 in mammalian cells (Fig. 2). The lack of an RA domain in RINL might cause a lower GEF activity, since interactions of RIN1 and RIN2 with GTP-bound Ha-Ras via their RA domains have been reported to potentiate their GEF activities for Rab5 proteins [8], [11]. The GEF activity of RINL is low under basal conditions but might be upregulated dramatically by certain stimulators, and its SH2 domain might be responsible for this regulation. This hypothesis is supported by reports that EGF stimulation induces a rapid and transient activation of Rab5a [24], and RIN1 forms complexes with a number of RTKs via its N-terminal SH2 domain [9], [10]. Deletion of the SH2 domain in RIN3 significantly reduced its GEF activity for Rab5 and Rab31 [13]. Identification of upstream inducers will uncover the molecular mechanism by which the GEF activity of RINL is regulated.

Recent reports have suggested that Rab22 plays a role in the heterotypic fusion of transported vesicles with other organelles. CHO cells expressing Rab22 associate with early and late endosomes [25]. The Rab22/Q64L mutant, which lacks GTPase activity, causes a prominent morphological enlargement of both early and late endosomes. Meanwhile, Rab22 regulates the recycling of major histocompatibility complex class I (MHCI) from early endosomes to the plasma membrane [26]. Because Rab5 and Rab22 interact and colocalize with EEA1, an established marker of early endosomes [27], [28], the cooperative activation of Rab5 and Rab22 by RINL might facilitate intracellular traffic from the plasma membrane to late endosomes or recycling endosomes via early endosomes.

We identified that the SH2 domain of RINL and the PTB domain of odin are required for their interaction, and this interaction is independent of the phosphorylation status of odin. This independence is supported by the fact that the SH2 domain of RINL is missing the critical arginine residue (in the FLVR motif) that directly interacts with pTyr ligands. Furthermore, the PTB domain of odin belongs to the Dab-like subgroup, which can bind to peptides that are not tyrosine phosphorylated [15]. A substitution of this arginine residue in the SH2 domain is also found in human RIN2. RINL and RIN2 interact more strongly with odin than other RIN family members, and odin interacts with the EphA8 receptor. These results suggest that RIN2 and RINL might require adaptor molecules to interact with RTKs. To identify further interacting molecules, crystal structures of their SH2 domains should be performed.

We also found that RINL overexpression promotes the degradation of EphA8 in an odin-dependent manner (Fig. 4B and C). Another report showed that overexpressed odin interacts with EphA8 and protects it from ubiquitination by Cbl, following degradation stimulated by ephrin-A5 [18]. This report also showed that odin binds to ubiquitinated EphA8 more strongly than non-ubiquitinated EphA8 through its SAM domains [18]. We found that Rab5-GEF activity of RINL is not altered by ephrin-A5 stimulation in HEK293T cells transfected with EphA8-FLAG (data not shown). Therefore, it is likely that ephrin stimulation induces the ubiquitinated EphA8-odin complex to become internalized after its interaction with RINL at the plasma membrane. Understanding how RINL interacts with odin and EphA8 after ephrin-A5 stimulation would reveal the precise molecular mechanism by which EphA8 is degraded.

## Materials and Methods

### Antibodies and reagents

Monoclonal anti-FLAG (M2), anti-c-myc (9E10), and anti-V5 antibodies were purchased from Sigma. Monoclonal anti-phosphotyrosine (pY) and anti-actin antibodies were from Millipore. Monoclonal anti-transferrin receptor and polyclonal anti-HaloTag antibodies were purchased from Invitrogen and Promega, respectively. All other reagents were from commercial sources and of analytical grade. Anti-odin rat monoclonal antibody was raised against a synthetic peptide corresponding to 14 amino acids from the C-terminal region of human odin. Anti-RINL rabbit polyclonal antibody was raised against a recombinant protein corresponding to 266 amino acids from the N-terminal region of human RINL.

### Construction of expression vectors

pCMV-FLAG-RIN3, Rabex-5, and pCMV-myc-Rab5a, 5b, 5c, 21, 22, and 31 were constructed as described previously [6], [7]. RINL mutants lacking GEF activity, pCMV-FLAG-RINL-D453A/P457A and -Y494A/T497A, were generated by PCR-mediated mutagenesis. Rab3a, Rab7a, Rab11a, Rab22, and EphA8 were amplified from a human leukocyte cDNA library. Various deletion mutants of RINL and odin were amplified using PCR. HaloTag-odin was purchased from the Kazusa DNA Research Institute.

### RNA Interference

RNA interference-mediated RINL knockdown was performed by transfecting Stealth RNAi siRNA with Lipofectamine RNAiMAX (Invitrogen). The targeted sequence of human RINL was 5′-GCUGCACAGAAAGGAUCAUCCCAGA- 3′. After transfection, cells were cultured for 72 hours. The siRNA-resistant RINL construct was generated by PCR-mediated mutagenesis using primers 5′-ACCCAAGGGCCCAGGCCAACCT-3′ and 5′-GGTCTTTCCTATGAAGCGTCCGCCTG-3′.

### Cell culture and transfection

HEK293T, HeLa, and Neuro2a cells were purchased from the ATCC and maintained as described previously [6]. Cells were transfected with plasmid constructs using either LipofectAMINE 2000 (Invitrogen) or HEKFectin (Bio-Rad) according to the manufacturer's protocols.

### Immunoprecipitation and gel filtration analyses

Immunoprecipitation and immunoblot analysis were performed as described previously [6]. Gel filtration analysis was performed as described previously [7].

### Production of recombinant proteins

Sf9 cells were purchased from the ATCC. FLAG-RINL, RIN3, and Rabex-5 were purified from baculovirus-infected Sf9 cells with anti-FLAG M2 agarose beads as described previously [29]. GST-fused Rab5 (5a, 5b, and 5c), Rab21, Rab22, and Rab31 recombinant proteins were expressed in and purified from the cytoplasmic fraction of pGEX6P-1-transformed *E. coli* BL21-CodonPlus (DE3)-RIL (Stratagene) by glutathione Sepharose 4B resin (GE Healthcare).

### In vitro guanine nucleotide-binding assay

The GTPgammaS-binding assay was performed using the filter method as described previously [6]. Briefly, GST-Rab5, Rab21, Rab22, and Rab31 (2.0–3.5 pmol GTPgammaS-binding activity) were incubated with 1 µM [^35^S]GTPgammaS (20,000 cpm/pmol) at 30°C for the indicated times in the presence or absence of FLAG-RINL, RIN3, or Rabex-5 purified from baculovirus-infected Sf9 cells.

### Radiolabeling of nucleotides associated with the Rab family in intact cells and identification of nucleotide-bound forms

HEK293T were transfected with myc-Rab5 subfamily proteins and FLAG-Rab5-GEF proteins. Guanine nucleotides associated with myc-Rab5 subfamily were analyzed as described previously [12], [24].

### Yeast two-hybrid screening

A yeast two-hybrid assay was performed as described previously [6]. The yeast reporter-strain Hf7c was transformed with pGBT9-RINL using a lithium acetate-based method and grown in synthetic medium lacking tryptophan at 30°C for 3 days. The cells were transformed with the mouse brain MATCHMAKER cDNA library (Clontech) and plated on synthetic medium lacking leucine, tryptophan, and histidine at 30°C for 5 days. For interaction analyses, transformed yeasts were lifted onto filter papers, lysed in liquid nitrogen, and incubated with 5-bromo-4-chloro-3-indolyl beta-D-galactopyranoside.

## Supporting Information

Figure S1 *In vitro* GEF activity of the RINL for the Rab5 subfamily. (A–F) The purified GST-Rab5a (A, 3.5 pmol of alive GTPgammaS-binding activity), Rab5b (B, 2.5 pmol), Rab5c (C, 3 pmol), Rab21 (D, 2 pmol), Rab22 (E, 3 pmol), or Rab31 (F, 2 pmol) was incubated at 30°C with 1 µM [^35^S]GTPgammaS for the indicated times in the presence of 8 pmol of FLAG-RINL (filled squares), Rabex-5 (filled triangles) or FLAG peptide alone (open circles). The amounts of [^35^S]GTPgammaS bound to the Rab5 subfamily are illustrated as the functions of the incubation times.
(TIF)

Figure S2 RIN2 and RIN3 exhibit GEF activities for Rab22 *in vitro*. GST-Rab22 (2 pmol of alive GTPgammaS-binding activity) was incubated at 30°C with 1 µM [^35^S]GTPgammaS for the indicated times in the absence (Rab alone) and presence of 8 pmol of RIN1 (filled squares), RIN2 (filled diamonds), RIN3 (filled circles) or FLAG-Rabex-5 (filled triangles). No [^35^S]GTPgammaS-binding activity was detected in the fractions of the RIN family or Rabex-5 (data not shown).
(TIF)

Figure S3 Diagram of the structural features of the odin/Anks1a. The numbers represent the amino acid residues. cDNA coding 583–1150 amino acids of odin was identified to interact with RINL in beta-galactosidase assay by yeast two-hybrid system.
(TIF)

Figure S4 RINL interacts with odin independent of its GEF activity. Wild type and point mutants that lost GEF activities for Rab5 were transiently co-transfected with myc-odin into HEK293T cells. Cells lysates were immunoprecipitated with anti-FLAG antibody, followed by immunoblotting with anti-myc and anti-FLAG antibodies. Aliquots of total lysates were also immunoblotting with anti-myc antibody.
(TIF)

Figure S5 Diagrams of deletion mutants of RINL and odin. The numbers represent the amino acid residues.
(TIF)

Figure S6 EphA8 stably expressing in Neuro2A cells is degraded by the expression of RINL. Neuro2A cells stably expressing EphA8-HA are transfected with myc-mock, RINL/WT, or RINL/YT_AA for 24 hours, and total lysates from these cells were immunoblotted with antibodies as indicated.
(TIF)
