# Supplementary figures and images for: RINL, Guanine Nucleotide Exchange Factor Rab5-Subfamily, Is Involved in the EphA8-Degradation Pathway with Odin
Source: PLoS One. 2012 Jan 23;7(1):e30575. doi: 10.1371/journal.pone.0030575 (PMC3264577; doi:10.1371/journal.pone.0030575)

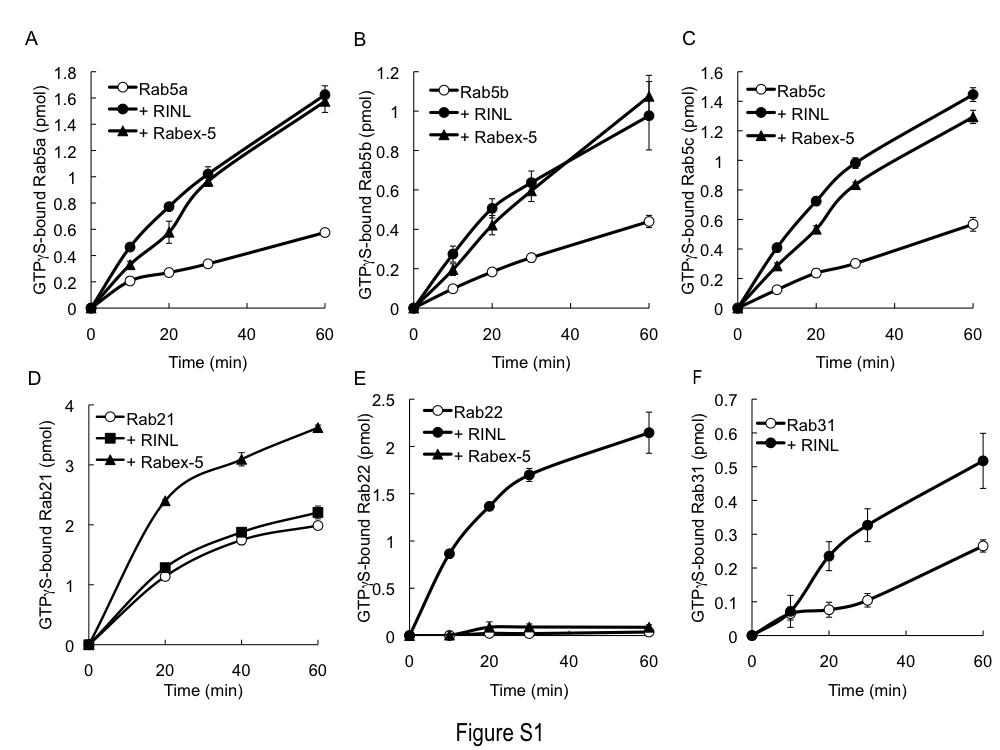

Supplement: Figure S1 — In vitro GEF activity of the RINL for the Rab5 subfamily. (A–F) The purified GST-Rab5a (A, 3.5 pmol of alive GTPgammaS-binding activity), Rab5b (B, 2.5 pmol), Rab5c (C, 3 pmol), Rab21 (D, 2 pmol), Rab22 (E, 3 pmol), or Rab31 (F, 2 pmol) was incubated at 30°C with 1 µM [35S]GTPgammaS for the indicated times in the presence of 8 pmol of FLAG-RINL (filled squares), Rabex-5 (filled triangles) or FLAG peptide alone (open circles). The amounts of [35S]GTPgammaS bound to the Rab5 subfamily are illustrated as the functions of the incubation times. (TIF) [file pone.0030575.s001.tif]

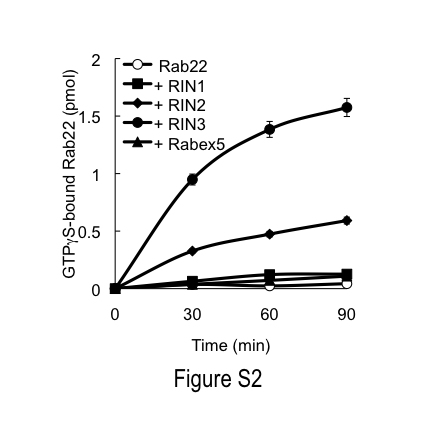

Supplement: Figure S2 — RIN2 and RIN3 exhibit GEF activities for Rab22 in vitro. GST-Rab22 (2 pmol of alive GTPgammaS-binding activity) was incubated at 30°C with 1 µM [35S]GTPgammaS for the indicated times in the absence (Rab alone) and presence of 8 pmol of RIN1 (filled squares), RIN2 (filled diamonds), RIN3 (filled circles) or FLAG-Rabex-5 (filled triangles). No [35S]GTPgammaS-binding activity was detected in the fractions of the RIN family or Rabex-5 (data not shown). (TIF) [file pone.0030575.s002.tif]

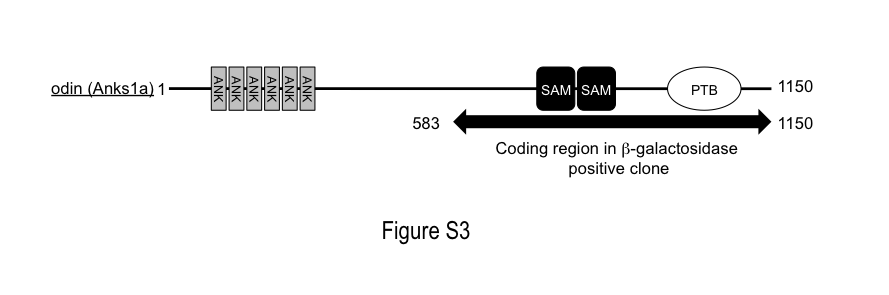

Supplement: Figure S3 — Diagram of the structural features of the odin/Anks1a. The numbers represent the amino acid residues. cDNA coding 583–1150 amino acids of odin was identified to interact with RINL in beta-galactosidase assay by yeast two-hybrid system. (TIF) [file pone.0030575.s003.tif]

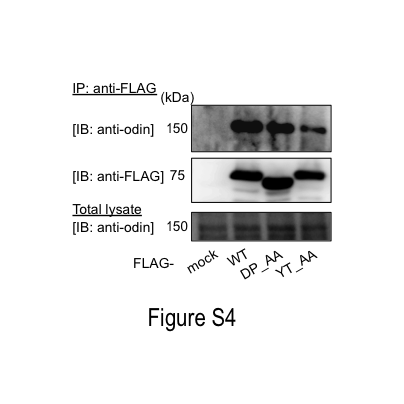

Supplement: Figure S4 — RINL interacts with odin independent of its GEF activity. Wild type and point mutants that lost GEF activities for Rab5 were transiently co-transfected with myc-odin into HEK293T cells. Cells lysates were immunoprecipitated with anti-FLAG antibody, followed by immunoblotting with anti-myc and anti-FLAG antibodies. Aliquots of total lysates were also immunoblotting with anti-myc antibody. (TIF) [file pone.0030575.s004.tif]

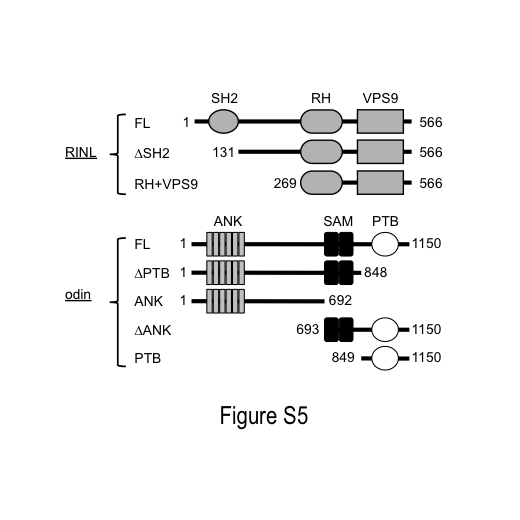

Supplement: Figure S5 — Diagrams of deletion mutants of RINL and odin. The numbers represent the amino acid residues. (TIF) [file pone.0030575.s005.tif]

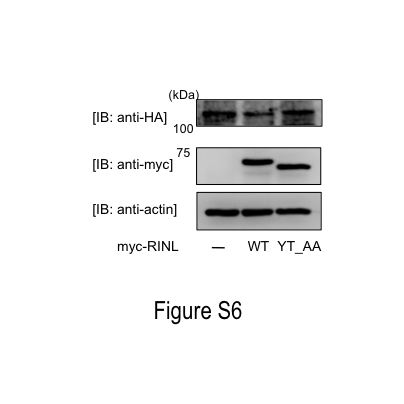

Supplement: Figure S6 — EphA8 stably expressing in Neuro2A cells is degraded by the expression of RINL. Neuro2A cells stably expressing EphA8-HA are transfected with myc-mock, RINL/WT, or RINL/YT_AA for 24 hours, and total lysates from these cells were immunoblotted with antibodies as indicated. (TIF) [file pone.0030575.s006.tif]
